# Surface adsorption and lubrication properties of plant and dairy proteins: A comparative study

**DOI:** 10.1016/j.foodhyd.2020.106364

**Published:** 2021-02

**Authors:** Morfo Zembyla, Evangelos Liamas, Efren Andablo-Reyes, Kewei Gu, Emma M. Krop, Ben Kew, Anwesha Sarkar

**Affiliations:** Food Colloids and Bioprocessing Group, School of Food Science and Nutrition, University of Leeds, Leeds, LS2 9JT, UK

**Keywords:** Mucin, Pea protein, Whey protein, QCM-D, Tribology, Lubrication

## Abstract

The aim of this work was to compare the surface adsorption and lubrication properties of plant and dairy proteins. Whey protein isolate (WPI) and pea protein isolate (PPI) were chosen as model animal and plant proteins, respectively, and various protein concentrations (0.1–100 mg/mL) were studied with/without heat treatment (90 °C/60 min). Quartz crystal microbalance with dissipation monitoring (QCM-D) experiments were performed on hydrophilic (gold) and hydrophobic polydimethylsiloxane (PDMS) sensors, with or without a mucin coating, latter was used to mimic the oral surface. Soft tribology using PDMS tribopairs in addition to wettability measurements, physicochemical characterization (size, charge, solubility) and gel electrophoresis were performed. Soluble fractions of PPI adsorbed to significantly larger extent on PDMS surfaces, forming more viscous films as compared to WPI regardless of heat treatment. Introducing a mucin coating on a PDMS surface led to a decrease in binding of the subsequent dietary protein layers, with PPI still adsorbing to a larger extent than WPI. Such large hydrated mass of PPI resulted in superior lubrication performance at lower protein concentration (≤10 mg/mL) as compared to WPI. However, at 100 mg/mL, WPI was a better lubricant than PPI, with the former showing the onset of elastohydrodynamic lubrication. Enhanced lubricity upon heat treatment was attributed to the increase in apparent viscosity. Fundamental insights from this study reveal that pea protein at higher concentrations demonstrates inferior lubricity than whey protein and could result in unpleasant mouthfeel, and thus may inform future replacement strategies when designing sustainable food products.

## Highlights

• Adsorption and lubrication properties of plant and dairy proteins were compared.
• Pea protein isolate (PPI) adsorbs to a large extent than whey protein isolate (WPI).
• Presence of mucin coating affects adsorption of both proteins.
• Lubrication properties of PPI declines at higher concentration as opposed to WPI.
• Heat treatment of proteins increase viscosity leading to enhanced lubrication.

## Graphical abstract

**Figure:**
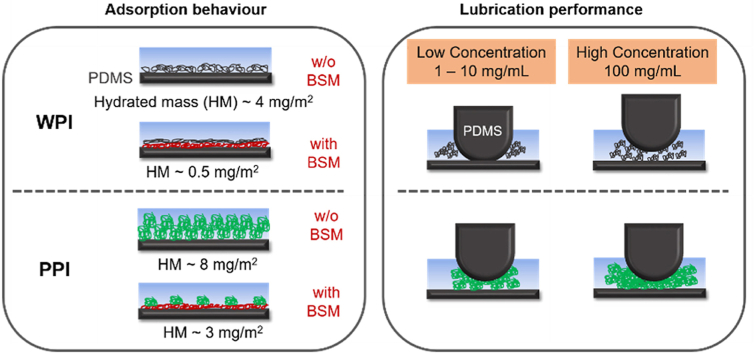

## 1 Introduction

The increasing number of overweight and obese people in the population has put a growing demand on the consumption of low fat foods. Consequently, there is significant research on designing fat replacers that mimic the physicochemical, mechanical and sensorial properties of fats in order to formulate low-fat or non-fat foods that are accepted by consumers. Among various fat replacers, whey proteins in various dairy products in native, heat-denatured or microparticulated forms (Fang, Shen, Hou, & Guo, 2019; Jørgensen et al., 2015; Olivares, Shahrivar, & de Vicente, 2019; Torres et al., 2018; Yilsay, Yilmaz, & Bayizit, 2006) have been extensively explored, however, their exact lubrication properties have attracted rare attention in literature to date.

With the rise in veganism and increased sustainability concerns, there has been increased interest towards use of alternative sources of proteins (Liu, Wang, Liu, Wu, & Zhang, 2018; Sun, Chen, Liu, Li, & Yu, 2015) to replace animal proteins. Consequently, plant proteins (*e.g.*, pea, soy) as alternatives to conventional animal proteins (*e.g.* casein, whey protein, gelatine) have gained significant research attention in recent years owing to the lower environmental footprints as compared to those associated with the production and consumption of the animal proteins (Jędrejek, Levic, Wallace, & Oleszek, 2016; Koneswaran & Nierenberg, 2008; Rodahl, Höök, Krozer, Brzezinski, & Kasemo, 1995; Zhang, Holmes, Ettelaie, & Sarkar, 2020). Although soy protein has often been considered as an alternative to the dairy counterparts (Shevkani, Singh, Kaur, & Rana, 2015), pea protein has been the preferred choice recently owing to a number of health benefits (Dahl, Foster, & Tyler, 2012), low cost, high abundance, as well as benefits from having a hypoallergenic and gluten-free status (Lan, Chen, & Rao, 2018). A recent study has investigated the use of pea protein as a replacement of milk proteins in food products and revealed that sensorial acceptability gradually decreased with increased levels of replacement by pea protein (Omrani Khiabanian, Motamedzadegan, Naghizadeh Raisi, & Alimi, 2020). However, the physical mechanism behind such sensorial difference between milk protein and pea protein was not well understood.

Pea protein exists as a complex mixture of protein aggregates (up to 36%), comprising of several proteins such as legumin (11 S), vicilin (7 S) and convicillin, with 11 S and 7 S subunits (Adal et al., 2017; Chen et al., 2019; Laguna, Picouet, Guàrdia, Renard, & Sarkar, 2017). There is an increased research interest to understand the structure-function similarity between whey and pea proteins in order to use cheaper pea protein to replace whey protein for sustainability purposes. Structurally, pea protein shows some similarity to whey proteins (comprising of globular *β*-lactoglobulin (*β*-lg) (Sarkar & Singh, 2016)) with respect to both these proteins comprising of largely globular fractions. However, the functional properties of pea protein are limited with respect to the whey counterpart owing to the limited aqueous solubility of pea protein (Adal et al., 2017; Zhang et al., 2020). Of a more relevant concern is that often pea proteins suffer from an unpleasant sensory perception, such as ‘astringency’ *i.e.* a dry or rough mouthfeel, and bitter off notes (Zeeb et al., 2018). Although astringency perception in pea protein has been linked to the tannins present in the pea protein mixture that chemically interact and precipitate with salivary proteins (Troszyńska, Amarowicz, Lamparski, Wołejszo, & Baryłko-Pikielna, 2006), a detailed physical mechanism behind the textural unpleasantness of pea proteins remains elusive in literature.

Recent progress in the field of oral tribology *i.e.* the study of friction and lubrication, has allowed the deciphering of the tribological mechanisms behind astringency (Sarkar, Andablo-Reyes, Bryant, Dowson, & Neville, 2019; Stokes, Boehm, & Baier, 2013). For example, sensory astringency in high-temperature short-time pasteurized as well as ultra-pasteurized milks has been shown to be positively correlated with friction coefficients in the mixed lubrication regime (Li, Joyner, Carter, & Drake, 2018). Such astringency was hypothesized to be associated with the heat-induced aggregation of whey proteins and casein in milk, which suggests that oral tribology can be a useful tool to understand the surface-related textural perception in pea proteins.

In addition to tribological analysis, quartz crystal microbalance with dissipation monitoring (QCM-D) is a highly sensitive technique that can measure adsorption in real-time. QCM-D has been successfully used in the past to provide powerful insights into nanoscale adsorption of proteins and polysaccharides on silicon-coated surfaces or on surfaces that mimic oral mucosa through incorporation of adsorbed layers of mucin or human salivary films (Glumac, Ritzoulis, & Chen, 2019; Kim, Weber, Shin, Huang, & Liu, 2007; Macakova, Yakubov, Plunkett, & Stokes, 2010; Stokes, Macakova, Chojnicka-Paszun, de Kruif, & de Jongh, 2011; Xu et al., 2020). For instance, investigating *β*-lg (the major fraction of whey protein) at different pH values on gold sensors demonstrated that greater adsorption occurred below the isoelectric point (Jachimska, Świątek, Loch, Lewiński, & Luxbacher, 2018). On the other hand, tribology studies showed that surfaces coated with *β*-lg had a much higher friction coefficient at pH 3.5 as compared to that at pH 7.0 (Çelebioğlu, Gudjónsdóttir, Chronakis, & Lee, 2016) and astringency was higher at the acidic pH (Ye, Streicher, & Singh, 2011). The results of sensory and friction studies demonstrated a clear relationship between foods' lubricating properties and sensation of roughness (Sarkar & Krop, 2019). Reduced lubrication, *i.e.* increased friction coefficient, has been typically associated with reduced sensation of creaminess and fattiness, and increased sensation of roughness.

Consequently, a fundamental question arises on whether friction coefficient and surface adsorption can be used as feasible *in vitro* approaches to decipher the fundamental mechanism by which proteins interact with oral surfaces. For example, understanding the variations in adsorbed mass and the viscoelastic properties of the resulting film derived by QCM-D may be correlated to lubrication data. There has been elegant progress in the field by using QCM-D as a complementary technique to tribological analsyis for salivary proteins, food-saliva interactions and polysaccharides (Stokes et al., 2011; Wang, Olarte Mantilla, Smith, Stokes, & Smyth, 2020; Xu et al., 2020; Yakubov, Macakova, Wilson, Windust, & Stokes, 2015). For instance, using polysachharides, Stokes et al. (2011) demonstrated that the product of velocity and lubricant viscosity (*Uη*) at the minimum friction coefficient *i.e.* at the junction between the mixed and hydrodynamic regimes in a Stribeck curve, was inversely correlated with the hydrated mass derived from QCM-D measurements.

In this direction, research on using QCM-D as a complementary tool to tribological and rheological analyses on plant proteins and comparison with animal proteins will advance our fundamental understanding behind the physical mechanisms underpinning the textural differences between these proteins. Therefore, the aim of this study was to compare the rheology, adsorption properties and the lubrication performance of the soluble fraction of pea and whey proteins, with or without heat treatment, using a combination of surface adsorption, tribology, rheology, surface wettability and other physicochemical characterization techniques. We have used the same concentration of the soluble fractions of pea and whey protein to compare their mechanical properties, as concentration is a common parameter used in product design when replacing animal proteins by plant proteins (Ainis, Ersch, & Ipsen, 2018; Omrani Khiabanian et al., 2020; Wu et al., 2020).

Polydimethylsiloxane (PDMS) surfaces were used to replicate the conditions found in the oral cavity. Although smooth PDMS surfaces cannot represent the soft, hydrophilic and textured oral surfaces (Sarkar, Andablo-Reyes, et al., 2019; Stokes et al., 2013), it is still the closest model available. In addition to hydrophilic (gold) and hydrophobic PDMS sensors, salivary mucin-coated PDMS sensors were employed to compare the interactions of pea and whey proteins with the salivary mucins at the surface level. For the oral tribology experiments, PDMS surfaces were employed and the friction coefficients were compared at different protein concentrations. We believe that this study provides a fundamental insight on how plant proteins differ from dairy proteins on adsorption and lubrication properties, which could aid towards the development of food products based on plant proteins.

## 2 Materials and methods

### 2.1 Materials

Commercial pea protein isolate (Nutralys S85XF) (PPI) with 85% protein content was kindly gifted by Roquette (Lestrem, France). Whey protein isolate (WPI) containing 96.5% protein was obtained from Fonterra (Palmerston North, New Zealand). The proteins were used without any purification. 4-(2-hydroxyethyl)-1-piperazineethanesulfonic acid (HEPES) buffer and NaCl were purchased from PanReac AppliChem (Germany) and Fisher Chemicals (UK), respectively. Bovine submaxillary mucin (BSM) was purchased from Sigma Aldrich (Dorset, UK) and used as to mimic human salivary mucins (Sarkar, Xu, & Lee, 2019). BSM was purified by dissolving in ultrapure water at 30 mg/mL followed by dialysis in a 100 kDa molecular weight cut-off membrane (Spectrum Laboratories, USA) against ultrapure water for a week and lyophilized (Xu et al., 2020). Polydimethylsiloxane (PDMS) (Sylgard 184, Dow Corning, Midland, MI, USA, base fluid and cross-linker (10:1 w/w)) was used for creating PDMS-coated QCM-D sensors. Mini-PROTEAN TGX Gels, ProtoBlue Safe Colloidal Coomassie G-250 stain and all sodium dodecyl sulphate polyacrylamide gel electrophoresis (SDS-PAGE) chemicals were purchased from Bio-Rad Laboratories, UK. All solutions were prepared with Milli-Q water (water purified by a Milli-Q apparatus, Millipore Corp., Bedford, MA, USA) with a resistivity of 18.2 MΩ cm at 25 °C. Ammonia solution (25%) and hydrogen peroxide solution (30%) were purchased from Fisher Chemicals (UK) and Sigma-Aldrich (Dorset, UK), respectively.

### 2.2 Methods

#### 2.2.1 Preparation of protein solutions

All the protein solutions were prepared by dissolving WPI, PPI or BSM (0.1–100 mg/mL) in 10 mM HEPES buffer and 10 mM NaCl and adjusted to the human salivary pH (pH 6.8). The solutions were allowed to hydrate for 2 h to ensure optimum dissolution. To create soluble fractions, WPI and PPI dispersions were centrifuged for 30 min at 4000 rpm at 20 °C (Fresco 21 centrifuge, Thermo Fisher Scientific, Germany) and the supernatants were carefully collected using a syringe and were used for characterization or thermally processed at 90 °C for 60 min using a water bath for the heat-treated (HT) samples, which are named HT WPI and HT PPI from here on, respectively.

#### 2.2.2 Solubility (%)

The protein content was measured using a DC protein assay kit (Bio-Rad Laboratories, Hercules, CA) on a UV–Vis Spectrophotometer with an absorption wavelength of 750 nm. The protein soluble fraction (%) was calculated by dividing the calculated concentration from the one used initially to prepare the protein dispersions. These soluble fractions were used for all characterization experiments except for sodium dodecyl sulphate-polyacrylamide gel electrophoresis (SDS-PAGE) where both untreated and the centrifuged fractions were used.

#### 2.2.3 SDS-PAGE

Sodium dodecyl sulphate-polyacrylamide gel electrophoresis (SDS- PAGE) under reducing conditions was used to determine the composition of protein in the initial (untreated) WPI and PPI solutions and supernatant after centrifugation (*i.e.* the soluble fraction) with or without heat-treatment (90 °C/60 min). Approximately, 65 μL of WPI or PPI solution were mixed with 25 μL of SDS loading buffer (62.5 mM Tris-HCl, pH 6.8, 2% SDS, 25% glycerol, 0.01% bromophenol blue) and 10 μL of dithiothreitol (DTT, of a final concentration of 50 mM), heated at 95 °C for 5 min. The SDS-PAGE was carried out by loading 10 μL of protein marker and 10 μL of these samples-SDS buffer mixtures in the Mini-PROTEAN 8–10% TGX Gels in a Mini-PROTEAN II electrophoretic unit (Bio-Rad Laboratories, Richmond, CA, USA). The resolving gel contained 16% acrylamide and the stacking gel was made up of 4% acrylamide. The running process was undertaken at 200 V for 22 min. After the run, the gel was stained for 2 h using Coomassie Blue solution, which consisted of 90% ProtoBlue Safe Colloidal Coomassie G-250 stain and 10% ethanol. The gel was then destained using Milli-Q water overnight and scanned using the ChemiDoc™ XRS+ with image Lab™ Software (Bio- Rad Laboratories, Inc, USA).

#### 2.2.4 Preparation of PDMS-coated QCM-D sensors

PDMS-coated sensors may represent a better approximation for human oral surfaces as compared to conventional gold-coated sensors (Macakova et al., 2010; Stokes et al., 2011; Xu et al., 2020). In addition, QCM-D using these PDMS surfaces serve as better comparison to the tribology data, which were also performed using PDMS tribopairs. For the preparation of PDMS-coated QCM-D sensors, 10 wt% PDMS in toluene solution was prepared and left to stir for 24 h. Then the solution was further diluted with toluene to 0.5 wt% which was again left to stir for 24 h. Silica-coated QCM-D sensors (QSX-303, Q-Sense) were immersed in RCA solution (5 parts of deionized water, 1 part of ammonia and 1 part of aqueous H_2_O_2_ (hydrogen peroxide, 30%)) at 80 °C for 15 min to remove any organic material and insoluble particles, followed by three cycles of sonication in ultrapure water for 10 min each cycle before drying using liquid nitrogen gas. Finally, 100 μL of 0.5 wt% PDMS solution was placed on the substrate and was spin-coated at 5000 rpm speed for 60 s.

#### 2.2.5 QCM-D measurements

The real-time adsorption behavior of proteins was measured by QCM-D (E4 system, Q-Sense, Sweden), described in details elsewhere (Glumac et al., 2019; Rodahl et al., 1995; Xu et al., 2020). QCM-D can simultaneously measure the shifts in frequency and dissipation at different overtones occurring during adsorption and provide wealthy information on the adsorption kinetics, mass, and viscoelasticity of the adsorbing film. To investigate the effect of surface chemistry on the multilayered film formation, hydrophilic gold-coated sensors (QSX-301, Q-Sense) and hydrophobic PDMS-coated sensors were used. Gold sensors were cleaned for 10 min under UV/ozone, followed by sonication in a 2% w/w sodium dodecyl sulphate solution for 15 min, rinsing and sonication in ultrapure water for 15 min, and 10 min under UV/ozone. The PDMS sensors were cleaned by 30 s immersion in toluene, followed by 30 s immersion in isopropanol, then 2 min immersion in ultrapure water, drying with nitrogen gas and letting the remaining solvent molecules evaporate for 2 h. All the solutions were supplied into QCM-D chamber containing the gold or PDMS sensors by a peristaltic pump with a flow rate of 100 μL/min at 25 °C. The first step was to inject the buffer solution until a stable baseline was observed. Subsequently, for the adsorption of WPI or PPI solutions on gold or PDMS surfaces, solutions were injected into the system for at least an hour, allowing the system to equilibrate, followed by rinsing in buffer solution for 30 min.

For adsorption to the salivary mucin-coated gold sensors, BSM was first injected into the system and left to adsorb for 1 h under the flow conditions. The surface was then rinsed with HEPES buffer for 30 min, followed by introduction and adsorption of WPI or PPI (0.1 mg/mL), the protein films were left to equilibrate for ~1.5 h, before rinsing again with HEPES buffer. The data were fitted using the Voigt model for viscoelastic solids (namely, “Smartfit Model”) by Dfind software (Q-Sense, Sweden) to obtain the mass of the hydrated protein layers. For improved visualization only the 5th overtone has been used in graphs. Each sample was measured in triplicates and means and standard deviations were reported.

#### 2.2.6 Dynamic light scattering experiments

The mean hydrodynamic diameter (*d*_H_) of WPI and PPI under different treatments (soluble fraction without or with heat treatment) was measured by dynamic light scattering at 25 °C via a Malvern Zetasizer Nano-ZS instrument (Malvern Instruments, Worcestershire, UK). Assuming particles to be spherical, the apparent particle diameter was calculated from the measured diffusion coefficient (*D*) via the Stokes- Einstein equation:

(1) $$d_{H}=\;\frac{k_{b}T}{3\pi \eta D}$$

where, *k*_*b*_ is the Boltzmann constant, *T* is the temperature and *η* is the viscosity of the solution. The size was measured at protein concentration of 0.1 mg/mL. The pH was adjusted to 6.8 by adding few drops of 0.1 M HCl or NaOH. One mL of solution was injected into a clean cuvette (PMMA, Brand Gmbh, Wertheim, Germany). The refractive index of proteins and the dispersion medium were set at 1.52 and 1.33, respectively. The absorbance of the protein was assumed to be 0.001. The hydrodynamic diameters (*d*_H_) were calculated by the cumulant analysis method of the autocorrelation function from the Zetasizer software. Each sample was measured in triplicates and each measurement was presented as the means and standard deviations of nine readings.

#### 2.2.7 Zeta-potential measurements

Zeta-potentials of WPI and PPI solutions under different treatments (soluble fractions without and with heat treatment) at pH 6.8 were measured in the standard folded capillary electrophoresis cells (DTS1070), using a Zetasizer Nano ZS instrument (Malvern Instruments Ltd., Worcestershire, UK). The concentration of WPI and PPI solutions was 0.1 mg/mL and the pH was adjusted to 6.8 by adding few drops of 0.1 M HCl or NaOH.

The instrument software was used to convert the electrophoretic mobility into ζ-potential values using the Smoluchowski (aqueous systems) or Hückel (non-aqueous systems) approximation. The ζ-potential was calculated from the measured electrophoretic mobility using the Henry's equation:

(2) $$\mu =\frac{\zeta \varepsilon_{r}\varepsilon_{o}f\left(\kappa \alpha \right)}{\eta }$$

where, *ζ* is the zeta potential, *κ* is the inverse of the Debye screening length, *α* is the particle radius, and *η* is the viscosity of the solvent. The value of *f* (*κα*) is determined by the medium, the electrolyte concentration, and the size of the proteins. In aqueous protein dispersions, where *κα* ≫ 1, *f* (*κα*) was 1 according to Smoluchowski approximation. Each sample was measured in triplicates and each measurement was presented as the means and standard deviations of nine readings.

#### 2.2.8 Static water contact angle measurements

The static water-contact angles were measured using a drop-shape analysis device (OCA 25, Dataphysics UK). After film formation by submerging the PDMS-coated crystals in the protein solutions (for 1.5 h) and then to the buffer (for 30 min), the sensors were air-dried with liquid nitrogen and kept in the temperature-controlled chamber (temperature set at 25 °C) of the drop-shape analysis device for static water contact angle measurements. The temperature of the experiments was set at 25 °C to reduce the droplet evaporation. Subsequently, 3 μL of MilliQ water was dispensed on the surfaces by the computer-controlled automatic liquid system. The averaged static water contact angle was then determined by the values of the right and left contact angles of the droplet, which was estimated from the image observed by a digital camera. Each measurement was performed in triplicate at different locations on the sensors.

#### 2.2.9 Shear rheology

Steady shear viscosity was measured using a commercial rotational rheometer Kinexus Ultra+ (Malvern, UK) equipped with a 50 mm diameter parallel plate geometry. The gap was fixed at 1.0 mm and the experimental temperature was kept at 37 °C to mimic the oral conditions. Shear viscosity was measured at a range of shear rates from 0.1 to 1000.0 s^−1^. Measurements were carried out in triplicates and the results presented are the arithmetic average of the independent readings.

#### 2.2.10 Soft tribology

Friction coefficients between compliant rolling/sliding surfaces were measured using a Mini Traction Machine MTM2 from PCS instruments (UK). The testing set-up was a ball (19.0 mm diameter) on disc contact, with both surfaces made of silicone (PDMS) with a Young's modulus of 2.4 MPa and average surface roughness of R_a_ ~50 nm. In order to mimic tongue/palate contact working conditions, temperature and contact normal force were fixed at 37 °C and 2.0 N respectively. The relative motion of rolling/sliding surfaces is commonly represented by the entrainment speed U, which for a ball on disc contact is obtained as $U=\frac{\left(u_{B}+u_{D}\right)}{2}$, where *u*_*B*_ and *u*_*D*_ are the ball and disc linear speeds at the contact point, respectively. Contributions to motion by either rolling or sliding were quantified by the rolling/sliding ratio (SRR) defined as $SRR=\frac{\left|u_{B}-u_{D}\right|}{\left(u_{B}+u_{D}\right)}$. Following current standards on mimicking oral conditions, the SRR was fixed at 50% and entrainment speed was swept from 0.3 to 0.003 m s ^−1^.

#### 2.2.11 Statistical analysis

Significant differences between samples were determined by one-way ANOVA and multiple comparison test with Tukey's adjustment performed using SPSS software (IBM, SPSS statistics) and the level of confidence was 95%.

## 3 Results and discussion

### 3.1 Physicochemical characteristics and composition of WPI and PPI dispersions

The final concentration and solubility of WPI and PPI (soluble fraction *i.e.* the supernatant after centrifugation) with or without heat treatment are shown in Table 1 (see Supporting Information, Table S1 for results of un-centrifuged samples). The concentration of the soluble fraction of WPI was 0.1 mg/mL where it showed 100% solubility, which is expected due to a large proportion of surface hydrophilic residues in the globular whey proteins (Lee, Morr, & Ha, 1992). However, upon thermal treatment, the concentration and water-soluble fractions in WPI decreased slightly (0.09 mg/mL and 90%, respectively) (Table 1). This is expected due to the unfolding of the previously buried hydrophobic groups and sulfhydryl/disulfide exchange chain reactions that take place between the exposed cysteine residues resulting in aggregation, and consequently, reduction in protein solubility (Dissanayake & Vasiljevic, 2009; Torres, Murray, & Sarkar, 2017).

**Table 1:** Final concentration, protein soluble fraction, size, PDI and ζ-potential of 0.1 mg/mL of water-soluble fractions (*i.e.* supernatant after centrifugation) of WPI and PPI dispersions at pH 6.8 with or without heat treatment. Samples with the same superscript letter do not differ significantly (*p > 0.05*) according to Tukey's test.

Samples	Final concentration (mg/mL)	Protein soluble fraction/%	*d*_H_ (nm)	PDI	*ζ*-potential (mV)
WPI	0.1 ± 0.02 ^a^	100	217.8 ± 27.3 ^d^	0.42 ± 0.08 ^f^	−20.2 ± 0.7 ^g^
HT WPI	0.09 ± 0.020 ^a^	90	174.7 ± 34.9 ^d^	0.34 ± 0.02 ^f^	−18.4 ± 1.0 ^g^
PPI	0.07 ± 0.01 ^b^	77.7 ^c^	239.0 ± 52.7 ^d^	0.55 ± 0.01 ^f^	−21.0 ± 1.1 ^h^
HT PPI	0.07 ± 0.01 ^b^	77.7 ^c^	132.0 ± 4.0 ^e^	0.29 ± 0.01 ^f^	−21.4 ± 3.1 ^h^

On the other hand, the soluble fraction of PPI was 0.07 mg/mL (the initial concentration before the removal of the non-soluble fraction was 0.1 mg/mL, see Supporting Information, Table S1), in other words, the solubility was ~77.7% indicating presence of a significant amount of aggregated and non-water soluble PPI fractions, which were removed during the centrifugation step (Adal et al., 2017). Heat treatment at 90 °C did not show any change to the concentration or protein solubility of the PPI dispersion.

The size, PDI and ζ-potential of WPI, HT WPI, PPI and HT PPI at pH 6.8 are shown in Table 1. After the centrifugation step (see Supplementary Table 1), the soluble fraction of WPI had a hydrodynamic diameter (*d*_H_) of ~218 nm and polydispersity index (PDI) ~ 0.4, which was statistically similar to that of PPI (*d*_H_ ~ 279 nm and PDI ~ 0.5) (*p < 0.05*). This suggests that even in the soluble fractions, the complex mixture of proteins, *i.e.* WPI or PPI, did not exist as monomers but as some sort of oligomers and loose aggregates (Adal et al., 2017; Loveday, Ye, Anema, & Singh, 2013). The size of WPI and PPI soluble fractions after heat treatment at 90 °C reduced significantly (*p < 0.05*) to ~175 and 132 nm for WPI and PPI, respectively. The PDI value also decreased, indicating that after heating, the protein oligomers were rather uniform in size. The *ζ*-potential of both WPI and PPI dispersions at pH 6.8 was almost the same at −20 mV.

**Fig. 1:**
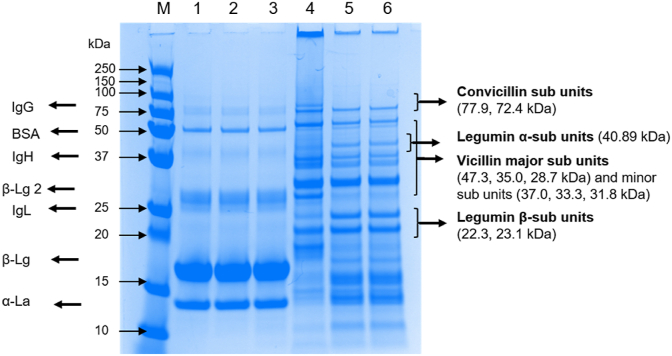
Sodium dodecyl sulphate polyacrylamide gel electrophoresis (SDS-PAGE) of untreated and soluble fractions of WPI, HT WPI, PPI and HT PPI. Protein concentration was 1 mg/mL. Lane 1–6 represents: (1) WPI (untreated fraction), (2) WPI (soluble fraction), (3) HT WPI, (4) PPI (untreated fraction), (5) PPI (soluble fraction) and (6) HT PPI. Lane (M) represents the molecular weight marker of 10–250 kDa molecular weight range.

In order to determine any differences in the protein composition between the untreated (*i.e.* the uncentrifuged) and the soluble fractions of WPI and PPI without and with heat treatment, SDS-PAGE was performed. As shown in Fig. 1 (lanes 1–3), WPI contains two major bands assigned to monomers of *α*-lactalbumin (*α*-la, 10 kDa) and *β*-lactoglobulin (*β*-lg, 18 kDa), two minor bands attributed to the dimers of *β*-lg (34 kDa) and bovine serum albumin (BSA, 50 kDa) and four faint bands showing presence of lactoferrin (LF, 75 kDa), Immunoglobulin G (IgG, 211 kDa), Immunoglobulin lambda locus (IgL, 20 kDa) and Immunoglobulin heavy locus (IgH, 37 kDa), which are in line with previous reports (Zhu, Damodaran, & Lucey, 2008). Centrifugation to remove the non-soluble fractions and/or thermal treatment did not incur any visible changes in the protein bands, indicating that different treatments (centrifugation or heat treatment) did not affect the composition of WPI.

On the other hand, pea protein showed legumin (11 S), vicillin (7 S) and albumins (2 S), with the most abundant globulins being 11 S and 7 S (Fig. 1) in line with previous reports (O'Kane, Vereijken, Gruppen, & Van Boekel, 2005). Untreated pea protein fractions (*i.e.* without the centrifugation step) and without heat treatment showed three sets of protein subunits, *i.e.* convicillin (72.4–77.9 kDa), vicillin (28.7–47.3 kDa) and legumin (22.3–23.1) subunits (Fig. 1, Lanes 4–6), which are in line with previous studies (Adal et al., 2017; Laguna et al., 2017; Zhang et al., 2020). However, centrifugation showed that some of the water-insoluble bands of the PPI, indicated by higher molecular weights, disappeared or the intensity of the bands became very weak suggesting removal of those fractions during the centrifugation step. From here on, only soluble fractions were employed for further characterization and concentrations of 0.1–100 mg/mL of soluble PPI or WPI were used.

### 3.2 Adsorption characteristics on gold and PDMS surfaces

**Fig. 2:**
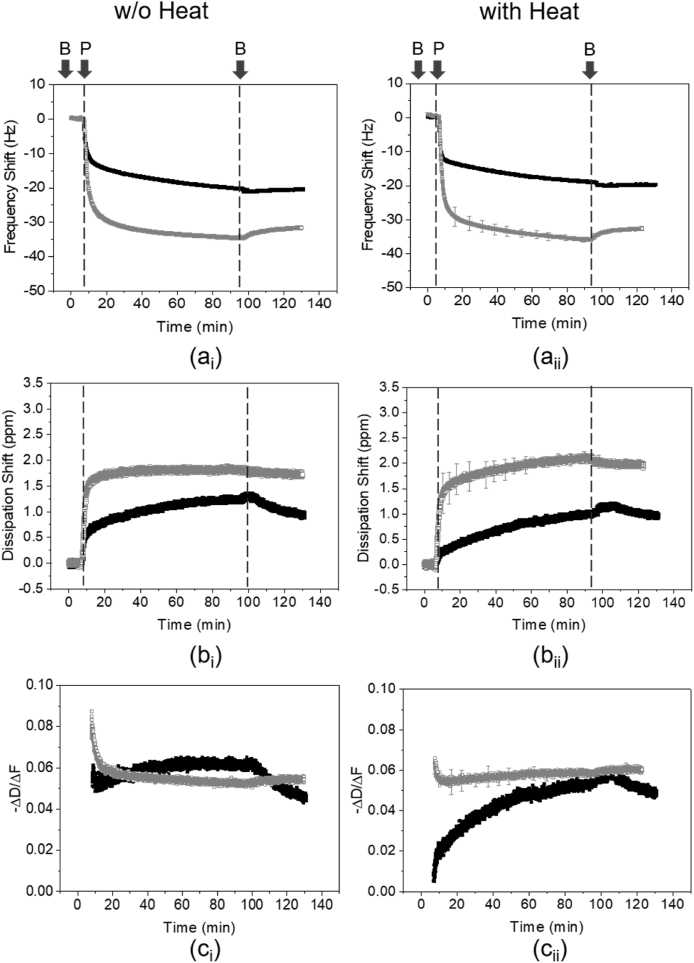
Frequency shift (a), dissipation shift (b) and *–ΔD/Δf* (c) obtained (5th overtone shown), as a function of time, of 0.1 mg/mL WPI (■) and PPI (□) on PDMS-coated surface without (i) or with (ii) heat treatment at 90 °C. B and P indicate the point of addition of buffer and protein, respectively. Error bars represent standard deviations.

QCM-D was used to record the frequency and dissipation shifts as a function of time in order to monitor the adsorption of proteins on gold, PDMS and mucin-coated PDMS surfaces. Fig. 2 shows the QCM-D results for the adsorption of the soluble fraction of PPI, HT PPI, WPI or HT WPI (at a concentration of 0.1 mg/mL) on hydrophobic PDMS-coated sensors. The trend of the plots for both WPI and PPI (without or with heat treatment) was very similar, indicating a rapid decrease in the frequency signal mirrored by a simultaneous dissipation increase when PPI or WPI was introduced, which is associated with fast adsorption as proteins arrive on an empty hydrophobic surface. Subsequently, protein adsorption continued to occur at a slower rate until adsorption-desorption equilibrium was reached, as indicated by a plateau in the frequency and dissipation signals for both proteins. Eventually, rinsing with buffer resulted in minor changes in frequency and dissipation of WPI system demonstrating very little desorption of the protein layer, whilst the frequency shift of the PPI system was larger on buffer rinsing. This effect is associated with the removal of loosely bound proteins from the surface, and the remaining of strongly bound protein molecules to the PDMS surface. Following rinsing with buffer, PPI revealed a frequency shift (Fig. 2a_i_ and 2a_ii_) of −30 and −35 Hz for non-heated and heated systems, respectively, while the dissipation shift (Fig. 2b_i_ and 2b_ii_) was approximately 1.7 ppm (non-heated) and 2 ppm (heated). On the other hand, for both non-heated and heated systems, the frequency shift and dissipation shift for WPI was approximately −20 Hz and 1 ppm, respectively. These results reveal a higher adsorption of PPI on the PDMS surface as compared to WPI, irrespective of heat treatment.

The parameter *−ΔD/Δf* (where *ΔD* and *Δf* are changes in dissipation and frequency, respectively) is commonly used to indicate the film properties; a higher *–ΔD/Δf* is often associated with a more viscous/less elastic film and vice versa (Ash et al., 2014; Madsen et al., 2016; Veeregowda et al., 2012; Xu et al., 2020). Fig. 2c_i_ and 2c_ii_ demonstrate that following rinsing with the buffer, PPI formed a slightly more viscous film than WPI. Furthermore, HT PPI was slightly more viscous as *–ΔD/Δf* increased (Fig. 2c_ii_). On the other hand, the viscoelastic properties of the adsorbed WPI film after rinsing were not affected by the thermal treatment.

Similar experiments were undertaken on gold sensors for non-heated and heated soluble WPI and PPI systems (see ~ Supporting Information, Fig. S1). In all the cases, the frequency shift (see ~ Supporting Information, Fig. S1a_i_ and S1a_ii_) in PPI after adsorption (−35 Hz for both non-heated and heated systems) was higher than WPI (−30 and −15 Hz for non-heated and heated systems, respectively). Furthermore, WPI forms a much more viscous film on hydrophilic gold sensors as opposed to that found on hydrophobic PDMS sensors (Fig. 2c_i_ and 2c_ii_), while PPI forms films of similar viscoelastic properties on both surfaces.

To examine if protein binding to the surface had undergone any structural rearrangements during the adsorption process, a plot of *ΔD* vs *Δf* (see Supporting Information, Fig. S2) was prepared and any change in the slope was determined. In such a plot, a relative small slope (*i.e.*, small dissipation gain for a given frequency shift) characterizes rigidly adsorbed layers, while a larger slope (*i.e.*, high dissipation for a given frequency shift) is representative of a soft viscoelastic layer (Teo et al., 2016). Figs. S2a and S2b revealed that both PPI and HT PPI samples displayed a simple behavior with a linear *ΔD/Δf* relation. In other words, the viscoelastic properties of the film were not changing during the adsorption. In contrast, WPI samples (with or without heat treatment) showed a more complex behavior, where the slope (*ΔD/Δf*) changed as a function of frequency shift during the adsorption. This was more apparent in the HT WPI sample, where the behavior of the slope consisted of two parts: an initial part exhibiting very low dissipation as compared to the slope of PPI sample, and a second part with an increasing *ΔD/Δf* value resulting in higher slope than PPI. Such change in the slope reveals that the structural conformation of the protein layer is altered during the adsorption process, leading to a less compact and softer adsorbed protein film (Dolatshahi-Pirouz et al., 2008). Consequently, heat treatment alters the adsorption characteristics of WPI, resulting to the formation of a compact (rigid) film on the substrate at the initial stages of the adsorption. As adsorption progresses, unfolding of protein molecules and increase of hydration lead to the formation of a softer film.

### 3.3 Adsorption characteristics on salivary mucin-coated surfaces

**Fig. 3:**
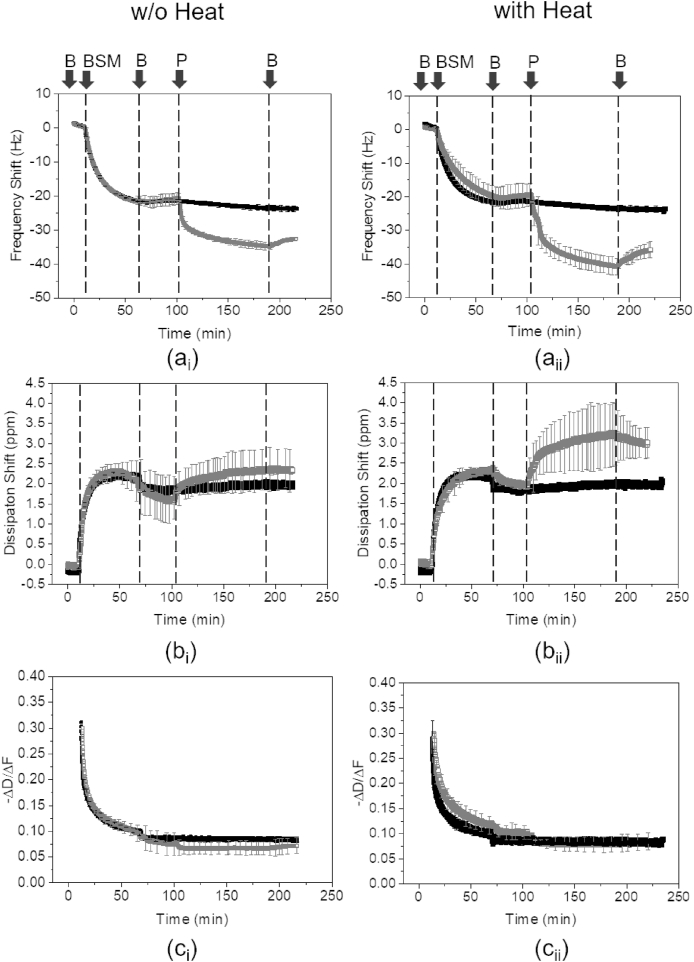
Frequency shift (a), dissipation shift (b) and *–ΔD/Δf* (c) obtained (5th overtone shown), as a function of time, of 1.0 mg/mL BSM followed by addition of 0.1 mg/mL WPI (■) and PPI (□) on PDMS-coated surface without (i) or with (ii) heat treatment at 90 °C. B, BSM and P indicate addition of buffer, BSM and protein, respectively. Error bars represent standard deviations.

Now we shift our focus to more orally relevant surfaces *i.e.* salivary mucin-coated surfaces (Xu et al., 2020) and compare the adsorption properties of soluble PPI versus WPI with or without heat treatment. The changes in frequency and dissipation during BSM and subsequent protein adsorption, with or without heat treatment, to a PDMS surface are shown in Fig. 3. Initially, introduction of BSM (1 mg/mL) resulted in fast adsorption onto the PDMS surface, indicated by a swift decrease in frequency (−20 Hz, Fig. 3a_i_ and 3a_ii_) and an increase in dissipation (~2.5 ppm, Fig. 3b_i_ and 3b_ii_), which is in agreement with previous report (Xu et al., 2020). Introduction of the buffer resulted in a small increase in frequency and a decrease in the dissipation due to rinsing away weakly bound BSM molecules. It is worth remembering that BSM did not undergo any heat treatment and, thus, similar behavior was observed in heat-treated and non-heat-treated systems.

Subsequent introduction of WPI and PPI on the adsorbed BSM layer revealed differences in their adsorption properties. On one hand, introducing PPI resulted in an immediate decrease in frequency, accompanied by an increase in dissipation, indicating rapid adsorption to the BSM-coated substrate, while rinsing with buffer removed some weakly adsorbed PPI molecules. The resultant relative frequency shift as compared with BSM (Fig. 3a_i_ and 3a_ii_) was approximately −15 Hz and −20 Hz without and with heat treatment, respectively, while the relative dissipation shift (Fig. 3b_i_ and 3b_ii_) was approximately 0.5 ppm and 1.0 ppm without and with heat treatment, respectively. On the other hand, introduction of WPI resulted only in a slight relative decrease in frequency (−5 Hz) while the dissipation remained the same. Subsequent rinsing with buffer resulted in minor changes in frequency and dissipation, indicating very little desorption of the protein layer. HT PPI resulted in increased adsorption, while HT WPI did not alter its adsorption characteristics on BSM-coated surfaces. Furthermore, as shown in Fig. 3c_i_ and 3c_ii_, the viscoelastic properties of the resulting WPI and PPI films were similar. The *ΔD* vs *Δf* plots of BSM and WPI or PPI adsorption (with or without heat treatment) on the BSM-coated PDMS sensor (Figs. S2c and S2d, Supporting Information) exhibited a linear relationship and revealed similar characteristics for both protein films. Although a stable baseline was achieved after incorporation of mucin and subsequent buffer rinsing, some exchange occurring between BSM layer underneath and the following dietary protein layers cannot be ignored, which needs further investigation in the future.

### 3.4 Hydrated mass on gold and PDMS surfaces

Table 2 shows the total hydrated mass of soluble WPI and PPI (~0.1 mg/mL) before and after heat treatment on gold, PDMS and mucin-coated PDMS surfaces. As shown, PPI exhibited a slightly larger adsorption on gold surface as compared to WPI. Although heat treatment reduced the adsorbed mass of PPI marginally , the impact on WPI was significant and the adsorbed mass reduced by half (see Supporting Information, Fig. S3). The surface adsorption rate for non-heated PPI on gold was approximately 2.5 mg m^−2^ min^−1^ for both PPI and HT PPI (Supplementary Table S2). Adsorption rate was higher for non-heated WPI (4.3 mg m^−2^ min^−1^) and upon heat treatment, the value was two-fold higher than PPI (4.9 mg m^−2^ min^−1^). On PDMS surfaces, PPI mass adsorption was significantly higher (*p < 0.05*) as compared to WPI, irrespective of heat treatment, while heat treatment resulted in increased hydrated adsorbed mass in both proteins though not significantly (*p > 0.05*). The adsorption rate for PPI did not change significantly upon adsorption on PDMS surfaces as compared to gold surfaces, with the rate being equal to 3.0 mg m^−2^ min^−1^ for both heated and non-heated PPI (see Supplementary Table S2). However, the initial rate of adsorption of WPI on PDMS was significantly decreased both for non-heated (0.7 mg m^−2^ min^−1^) and heated WPI (2.4 mg m^−2^ min^−1^). The results indicate WPI has a slight preference on hydrophilic gold surface while PPI prefers hydrophobic PDMS surfaces, which can be attributed to a more hydrophilic WPI molecule as compared to PPI. Furthermore, while heat treatment does not have a significant impact on PPI, it causes structural changes on WPI that result in three-fold higher adsorption on PDMS, indicating that heat treatment in HT WPI caused rearrangement of the WPI molecular structure leading to exposure of hydrophobic moieties that interact with the PDMS surface.

**Table 2:** Mean hydrated mass of BSM and total hydrated mass of WPI and PPI (heated or non-heated) after the adsorption onto gold or PDMS surface, latter in presence or absence of mucin coating. Samples with the same superscript letter do not differ significantly (*p > 0.05*) according to Tukey's test.

QCM-D sensors	Sample	Hydrated mass of BSM (mg/m^2^)	Total hydrated mass (mg/m^2^)
On Gold	WPI	–	8.89 ± 0.34 ^a^
	HT WPI	–	4.44 ± 0.26
	PPI	–	9.40 ± 0.37 ^a^
	HT PPI	–	9.16 ± 0.11 ^a^
On PDMS	WPI	–	4.30 ± 0.36 ^b^
	HT WPI	–	5.25 ± 0.11 ^b^
	PPI	–	8.41 ± 0.2 ^c^
	HT PPI	–	9.48 ± 1.02 ^c^
On PDMS	BSM + WPI	6.43 ± 0.01 ^d^	6.99 ± 0.1 ^f^
	BSM + HT WPI	5.60 ± 0.01 ^e^	6.07 ± 0.2 ^f^
	BSM + PPI	6.38 ± 0.30 ^d^	9.65 ± 0.12
	BSM + HT PPI	6.11 ± 0.24 ^d, e^	10.93 ± 0.61

Regarding the systems where BSM adsorbed first on PDMS surface, the initial adsorbed mass of BSM was approximately ~6 mg/m^2^ (Table 2). Adsorption of WPI and PPI on pre-coated surfaces with BSM was lower than on bare gold and PDMS surfaces, suggesting that the presence of BSM hindered adsorption of large quantities of WPI, PPI or HT WPI, HT PPI due to repulsive interactions between negatively-charged BSM (Xu et al., 2020) and negatively-charged proteins (Table 2) at orally relevant pH. Nevertheless, in a comparative sense, adsorption of WPI or HT WPI on the already adsorbed BSM layer resulted in only a small increase in adsorbed mass (~0.5 mg/m^2^), while the adsorption of PPI or HT PPI on BSM showed a higher mass adsorption (~3–4 mg/m^2^) (*p < 0.05*). In summary, it can be concluded that PPI adsorbs to a much larger extent as compared to WPI, both on bare and on salivary mucin-coated surfaces.

### 3.5 Wettability

**Fig. 4:**
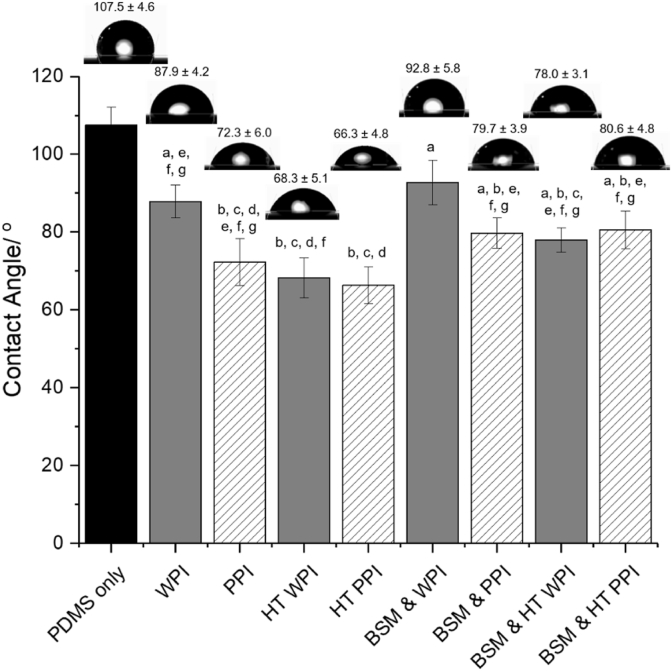
Mean static water contact angle of 0.1 mg/mL of WPI, HT WPI, PPI and HT PPI with and without the presence of BSM on the PDMS-coated surface. Error bars represent standard deviations. Samples with the same alphabet do not differ significantly (*p > 0.05*) according to Tukey's test.

In order to understand further the surface adsorption behavior, the static water contact angle was measured before and after adsorption of the protein films onto the hydrophobic PDMS surfaces (Fig. 4). As expected, the measured contact angle of the PDMS-coated sensor was ~107.5° indicating a hydrophobic surface (Sarkar, Kanti, Gulotta, Murray, & Zhang, 2017; Xu et al., 2020). It was observed that all films became less hydrophobic after protein adsorption on the surface with PPI coating making the PDMS surface more wettable than the WPI-coating (~88 and ~72° for WPI and PPI, respectively) (*p < 0.05*). After heat treatment, the static water contact angle of WPI samples decreased significantly (68°), suggesting that the heat-induced unfolding of globular WPI allowed more efficient adsorption to PDMS surface whilst the contact angle for HT PPI did not change significantly (*p > 0.05*).

Coating the hydrophobic PDMS surfaces with BSM entailed a significant reduction in contact angle (97.5°, data not shown). The value is much higher than values reported previously (Sarkar et al., 2017; Winkeljann et al., 2020) as PDMS was not hydrophilized by O_2_-plasma treatment prior to the physisorption by BSM in the current study as opposed to the previous reports. On BSM-coated surfaces, WPI and PPI were slightly more hydrophobic than those without BSM and also more than BSM itself (Fig. 4). While heat treatment allowed the contact angle to decrease significantly only for WPI samples (from 93° to 78° before and after heat treatment, respectively), for PPI samples no significant change was observed due to heat treatment (~80°, before and after heat treatment). The wettability results (Fig. 4) are in close agreement with the QCM-D results (Fig. 2, Fig. 3) suggesting that the presence of negatively-charged BSM coating somehow hinders the easy adsorption of the subsequent dietary protein layers. Nevertheless, PPI makes the surface more hydrophilic as compared to WPI, irrespective of the presence of BSM coating (Fig. 4) due to higher adsorption of PPI to the PDMS as well as the mucin-coated PDMS surfaces (Fig. 2, Fig. 3 and Table 2).

### 3.6 Friction coefficients and viscosity

**Fig. 5:**
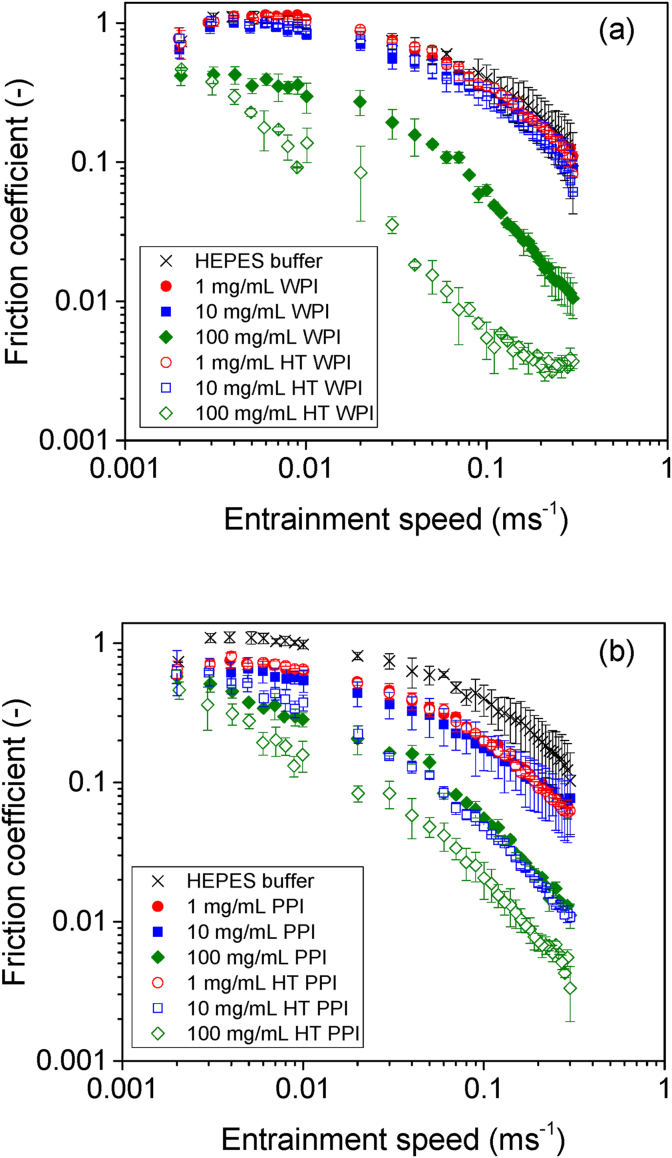
Friction coefficients of soluble fraction and heat-treated versions of (a) WPI and (b) PPI solutions as a function of entrainment speeds. Friction coefficients of buffer is also presented for comparison purposes.

It is crucial to understand how the surface adsorption properties of these proteins to PDMS surfaces may influence the tribological behavior when such proteins are under tribological stress, using PDMS-PDMS tribopairs. Fig. 5 shows the lubrication performance of non-heat-treated protein solutions and the HT counterparts, represented by the curves of friction coefficients as function of entrainment speeds. To facilitate the interpretation of friction coefficient curves, they are commonly divided into three different regimes determined by the lubricant involvement in the surface contact area. The boundary regime is commonly observed as a region in the curve with no speed dependence and the highest friction coefficient values. The high friction associated with this regime is attributed to the load being completely supported by the direct surface contact in the absence of a pressurized lubricant. The boundary regime is commonly observed at the lowest speeds and in simple cases of pure viscous lubrication, the extension of this regime within the speed experimental window is determined by the fluid viscosity.

Experimental observations reported in literature have shown that polysaccharides and salivary proteins are capable of reducing the friction in the boundary regime due to surface adsorption (Harvey, Yakubov, Stokes, & Klein, 2012; Stokes et al., 2011; Xu et al., 2020). This phenomenon is commonly known as hydration lubrication (Jahn & Klein, 2015). On increasing the entrainment speed, the boundary regime ends with the start of the mixed lubrication regime. In the mixed regime, viscous forces are capable of producing a discontinuous pressurized lubricant film partially separating the surfaces (Prakash, Tan, & Chen, 2013; Sarkar, Andablo-Reyes, et al., 2019; Stokes et al., 2013) and thus decreasing the contact friction relative to the boundary regime.

Since the hydrodynamic forces are proportional to the entrainment speed, the mixed lubrication regime is characterized by a monotonic decrease of friction on increasing the entrainment speed. Once a continuous pressurized film that is capable of completely separating the surfaces is formed, the soft contact enters in the elastohydrodynamic lubrication regime. The transition between the mixed and elastohydrodynamic regimes is observed as a minimum, after which the friction coefficient increases monotonically with increasing speed. In the case of complete ball on disc contact lubricated by Newtonian liquids, a mathematical expression that describes the friction coefficient in this regime is presented in equation (3) (De Vicente, Stokes, & Spikes, 2005):

(3) $$\mu =1.46{\underset{‾}{U}}^{0.65}{\underset{‾}{W}}^{-0.70}+SRR\left(3.8{\underset{‾}{U}}^{0.71}{\underset{‾}{W}}^{-0.76}+0.96{\underset{‾}{U}}^{0.36}{\underset{‾}{W}}^{-0.11}\right)$$

where *U* is the entrainment ($\underset{‾}{U}=\frac{U\eta }{E^{*}R^{*}}$), *W* is the applied load ($\underset{‾}{W}=\frac{W}{E^{*}{R^{*}}^{2}}$), *η* is the liquid viscosity, $E^{*}={\left(\frac{1–{v_{1}}^{2}}{E^{\prime }}+\frac{1–{v_{2}}^{2}}{E^{\prime \prime }}\right)}^{-1}$ and $R^{*}={\left(\frac{1}{R^{\prime }}+\frac{1}{R^{\prime \prime }}\right)}^{-1}$ are the reduced Young's modulus and reduced radius of the contact, respectively. Here $E^{\prime }$ and $E^{\prime \prime }$ are the elastic moduli of the tribo-surface materials of the ball and disc, respectively, and $R^{\prime }$ and $R^{\prime \prime }$are the radius of the ball and disc, respectively. Experimental results are described below using the aforementioned concepts.

Fig. 5a shows the friction curves obtained for non-heat-treated WPI and HT WPI solutions with concentrations varying from 1 to 100 mg/mL. Friction curves for solutions with protein concentrations of 1 and 10 mg/mL, either HT WPI or non-heat-treated WPI, are not significantly different from the HEPES buffer (*p > 0.05*). Thus, up to this concentration, the presence of protein did not show any benefits on lubrication. All these curves show a boundary lubrication regime with constant values of friction around 1.05, extending up to an entrainment speed value of about 0.01 m/s. On increasing the speed, the mixed lubrication regime is observed, with a friction coefficient down to a value of about 0.1 at the highest experimental speed of 0.3 m/s. On increasing the protein concentration up to 100 mg/mL, the boundary regime for the non-heat-treated WPI solution shows friction coefficient values of about 0.4. This signifies a bit more than a two-fold decrease in friction coefficients in comparison to the solutions with lower protein concentrations. The two-fold decrease is approximately constant in the entire experimental window, despite the transition from boundary to mixed lubrication regime. The earlier onset of mixed regime in case of 100 mg/mL WPI can be attributed to the surface adsorption of WPI layers to PDMS surfaces, as clearly shown by the hydrated mass adsorbed on the PDMS surface (Table 2) and nearly ~20° reduction in static contact angle versus PDMS surface (Fig. 4). Overall, this indicates that WPI above a certain concentration is capable of providing lubrication *via* surface adsorption similar to other biopolymers (Stokes et al., 2011).

**Fig. 6:**
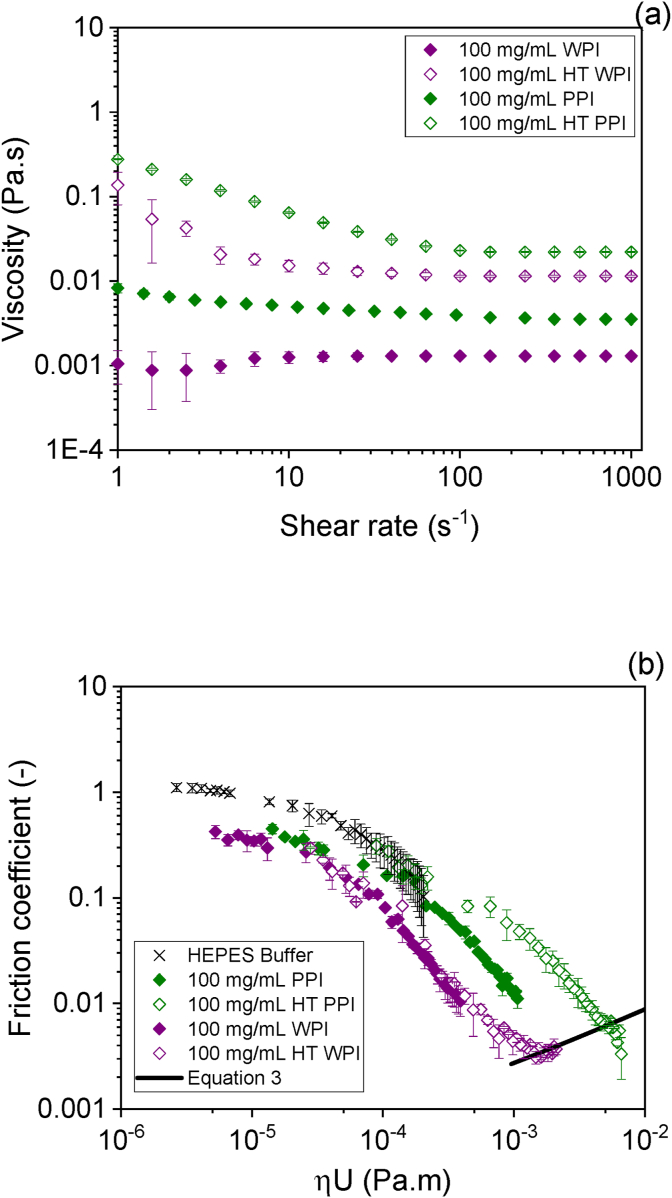
Shear viscosity (a) and friction coefficient (b) curves as a function of *ηU* of soluble fraction and heat-treated versions of WPI and PPI solutions at protein content of 100 mg/mL. The black continuous line represents the fitting using equation (3) to the elastohydrodynamic regime on the curve for the heat-treated WPI solution.

The HT WPI on the other hand showed lower friction coefficients even at lower concentrations (1–10 mg/mL) unlike non-heat-treated WPI. Such lubrication property of HT WPI corroborates three-times faster adsorption kinetics to PDMS as compared to non-heat-treated WPI (Supplementary Table S2) and a further ~20° reduction in static contact angle versus the WPI-coated PDMS surface (Fig. 4). In particular, HT WPI solution with 100 mg/mL concentration shows significantly lower friction coefficient in comparison to its non-heat-treated counterpart in the whole range of speeds. Another important difference is that the heat-treated solution shows no boundary regime, but only the mixed lubrication regime and the onset of the elastohydrodynamic lubrication regime are apparent. The shift to lower speeds for the onset of the mixed lubrication regimes is characteristic of an increase in the effective viscosity of the lubricating fluid, which is discussed further below (Fig. 6).

Fig. 5b shows the friction coefficients as function of entrainment speed obtained for non-heat-treated and HT PPI solutions. Unlike WPI (Fig. 5a), non-heat-treated PPI solutions with lower concentrations up to 10 mg/mL show the boundary and mixed lubrication regimes (Fig. 5a). These lower PPI concentrations (1–10 mg/mL) showed no significant differences (*p < 0.05*) in friction coefficient in the boundary region (U = 0.004 m/s) with values of 0.75 and 0.62 for the 1 and 10 mg/mL PPI solutions, respectively. This means that PPI solutions with at least 1 mg/mL can decrease the friction by about 40.0% as compared to the buffer. The two-times higher hydrated mass of PPI versus WPI (Table 2), the formation of a viscoelastic film of PPI (Fig. 2ai and 2ci) as well as the higher wettability (~15° reduction in static water contact angle versus WPI-coating on PDMS surface) aided in effective boundary lubrication even at very low protein concentrations. The ratio between friction coefficients for the buffer and the PPI solutions is mostly constant in the whole experimental window.

On increasing the PPI concentration to 100 mg/mL, only the mixed lubrication regime is observed with friction values starting at the level of the boundary regime obtained for the solutions with lower protein concentration. The shift of the onset of the mixed lubrication regime to lower speed indicates an increase of effective viscosity on increasing protein concentration. Focusing now on the HT PPI solutions, it is evident that heat treatment increases the viscosity of the solution consequently shifting the onset of the mixed lubrication regime relative to the non-heat-treated counterparts. However, heat treatment did not modify the friction coefficient values obtained in the boundary lubrication regime for HT PPI, which is also in line with limited change in adsorption versus non-heated counterparts (Table 2).

In the description of the lubrication performance of the dispersions presented above containing higher concentrations of proteins (100 mg/mL), it is clear that the definite role of viscosity needs to be elaborated. The product of viscosity and entrainment speed (*ηU*) can quantify the viscous forces acting on a lubricated contact. In the case of Newtonian fluids, representing friction coefficient as function of *ηU* results in the overlap of the friction curves obtained for fluids with different Newtonian viscosity (Bongaerts, Fourtouni, & Stokes, 2007). In the case of complex fluids exhibiting shear rate dependent viscosity, it is not straightforward to assume one single value of *η* to represent the viscous forces in the tribological limit. Nevertheless, previous work has demonstrated that the high shear rate limit viscosity (1000 s^−1^) is a good approximation to quantify the viscous forces of complex fluids in the tribological limit (Andablo-Reyes et al., 2019).

Fig. 6a shows the steady shear viscosity as a function of shear rate of non-heat-treated and heat treated protein solutions (100 mg/mL concentration). The non-heat-treated WPI solution is a Newtonian fluid with viscosity of 0.0013 Pa s. The non-heat-treated PPI solution has a slight shear thinning character and has a slightly larger viscosity in comparison to the WPI solution in the whole range of shear rates. This might be expected owing to the aggregates present in the PPI solution (see Table 1 for PDI) even after the centrifugation step, which were broken down in the direction of the flow. However, it is worth noting that at orally relevant speeds of 50–100 s^−1^, the viscosity values of PPI (0.0043 Pa s) and WPI (0.0013 Pa s) (Fig. 6a) were in the same order of magnitude and not significantly different (*p < 0.05*) in line with the size data in Table 1. Both, HT WPI and HT PPI are shear thinning fluids with higher viscosity values in comparison to their non-heat-treated counterparts (Fig. 6a).

As discussed above, the increase in viscosity due to heat treatment was apparent in the lubrication performance of the protein solutions. Here, the high shear rate viscosity was related to the viscous forces under lubrication conditions, *i.e.* friction coefficient as function of the product of *ηU* (Fig. 6b). The viscosity was determined using the values at a shear rate value of 1000 s^−1^. Values of *η* for heat-treated and untreated PPI solutions shown in Fig. 6b were 0.022 and 0.0042 Pa s, respectively. The heat-treated WPI solution shows the elastohydrodynamic lubrication at the highest entrainment speed (*U* > 0.15 m/s). This portion of the friction curve was used to estimate an effective *η* = 0.007 Pa s in the tribological limit by fitting equation (3), where the *η* was the only free parameter. The fitting is shown as a continuous black line in Fig. 6b. The overlap of the friction curves for PPI solutions with the buffer indicates that the capacity of PPI at higher concentrations (100 mg/mL) to lubricate relies on the viscosity of the solution. In Fig. 6b, friction coefficient curves for HT WPI and non-heat-treated WPI solutions overlapped, but they show friction coefficient values significantly lower in comparison to buffer. Lubrication of both PPI and WPI with protein concentrations of 100 mg/mL is provided by the combination of viscous (mixed and elastohydrodynamic regimes) and hydration forces (boundary regime), although hydration forces are more efficient in WPI in comparison to PPI. For both, WPI and PPI, the effect of heat treatment is shown to increase the effective viscosity in the tribological limit.

**Fig. 7:**
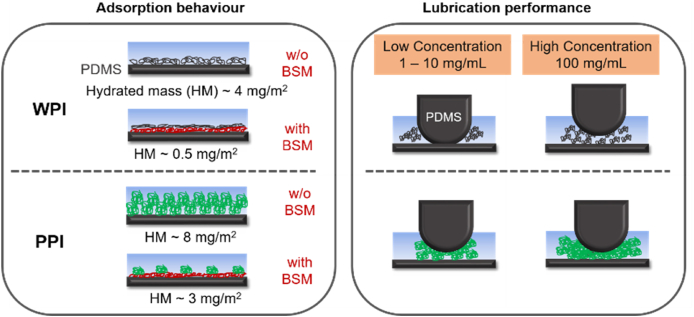
Schematic representation (not to scale) of the adsorption and lubrication behavior of WPI and PPI on PDMS surface, illustrating the effect of BSM on the adsorption behavior and hydrated mass (HM) of proteins and the impact of protein concentration on their lubrication performance.

To sum it all up (see schematic illustration in Fig. 7), non-heat-treated WPI and HT WPI with a protein content of up to 10 mg/mL show no clear benefit in lubrication. In contrast, at lower concentrations, PPI solutions (whether HT or non-heat-treated) can show significant reductions in friction at only 1 mg/mL in the boundary and mixed regimes. The reduction in friction for PPI at lower concentrations as opposed to WPI is associated with the increased adsorbed mass observed with QCM-D, wetting of the PDMS surfaces to a larger extent and decreasing the contact angle significantly (*p < 0.05*) (Fig. 2, Fig. 4, Table 1) leading to a higher initial rate of adsorption (Supplementary Table S2). This shows that PPI is capable to adsorb in larger extent (at least at low concentrations) in comparison to WPI. A direct relation between adsorption and boundary lubrication performance has been demonstrated for other biopolymers such as carbohydrates (Stokes et al., 2011). So, as shown in the schematic (Fig. 7), PPI forms a viscoelastic hydrated layer that can help in reducing friction between PDMS-PDMS contact surfaces at lower PPI concentrations.

On increasing WPI concentrations to 100 mg/mL (Fig. 7), WPI shows improved boundary lubrication as compared to PPI solution at the same concentration. PPI showed no improvement in boundary lubrication from lower concentrations as evident in Fig. 6b where tribology data was scaled with viscosity. It is clear from Fig. 6b, that the friction curves of PPI at higher concentrations (100 mg/mL) with or without heat treatment behave similar to that of buffer. This might be potentially attributed to PPI-PPI interaction leading to the formation of aggregates in the confinement as might be expected from high PDI (Table 1) and tendency of PPI to aggregate as reported previously (Adal et al., 2017). Such aggregates might have acted as particulates jamming the contact and rising the friction coefficients significantly as schematically illustrated in Fig. 7. Such jamming by protein-based particles has been observed previously for proteinaceous microgels (Sarkar et al., 2017). In other words, PPI behaves as a polymer at lower concentrations and acts as a boundary lubricant, but has a more particle-like behavior at higher concentrations increasing the friction coefficient. This polymer to particle-like transition as a function of concentration and its effects on tribology need further investigation in the future.

In summary, WPI requires larger concentrations in order to saturate the lubricated contact surfaces in comparison to PPI (Fig. 7), the latter can saturate the surface at concentration as low as 1 mg/mL. Thus, PPI might be suitable to replace lower concentrations (1–10 mg/mL) of WPI in food formulations due to effective lubrication performance associated with higher adsorption. However, such benefits are not achieved when the concentration of protein increases. The reason why the saturated film of WPI at higher concentrations decreases boundary friction in larger extent in comparison to PPI is not clear, but might be associated with the adsorption and viscoelastic properties of WPI films at high concentrations. Noteworthy, these higher concentrations could not be studied using QCM-D due to technical limitations, so it remains unclear what are the viscoelastic properties and film thickness of the PPI film at such higher concentrations. Finally, heat treatment does not alter significantly the capacity of protein to adsorb onto the PDMS surface or the viscoelastic properties of the adsorbed film (Fig. 2) but increases the lubrication capacity of the protein (at 100 mg/mL protein content) by increasing viscosity as evident in Fig. 6a.

## 4 Conclusions

In this work, we demonstrated for the first time the adsorption capacity and lubrication performance of PPI compared to WPI without or with heat treatment, on PDMS surfaces commonly used as model surfaces for oral lubrication studies. PPI adsorbs twice as much on the PDMS surfaces and forms a slightly more viscous film as compared to WPI, while heat treatment has minor impact on the amount of adsorbed hydrated mass of both PPI and WPI. QCM-D results also demonstrated that in the presence of salivary mucins, PPI is capable of adsorbing to a larger extent in comparison to WPI, forming films with similar viscoelastic properties. For concentrations up to 10 mg/mL, the larger adsorption capacity of PPI resulted in a significant reduction of friction in the boundary regime, which was not observed for WPI. However, upon increasing the concentration to 100 mg/mL, WPI improved the boundary lubrication showing better performance than the PPI solution with the same concentration, the latter showing improvement from lower concentrations only in the mixed regime due to the increase in viscosity. This shows that WPI requires larger concentrations in order to saturate the lubricated contact surfaces in comparison to PPI. In other words, replacement of large concentrations of WPI by PPI might have an adverse frictional consequence. Heat treatment of either PPI or WPI increased their viscosity, enhancing their mixed and elastohydrodynamic lubrication, but it did not affect the non-viscous (boundary) lubrication. Further work using friction force microscopy could provide a powerful insight in determining the lubricating properties of protein-coated PDMS surfaces at the nanoscale. Finally, the use of sensory panels to relate the adsorption and tribology parameters to specific mouthfeel attributes, is crucial to justify the importance of these *in vitro* techniques in research and the industrial community.

## CRediT authorship contribution statement

**Morfo Zembyla:** Writing - original draft, Methodology, Validation, Formal analysis, Investigation, Data curation, Writing - review & editing, Visualization, Project administration. **Evangelos Liamas:** Methodology, Validation, Formal analysis, Investigation, Data curation, Writing - review & editing, Visualization, Supervision. **Efren Andablo-Reyes:** Methodology, Validation, Formal analysis, Data curation, Writing - review & editing, Visualization. **Kewei Gu:** Formal analysis, Investigation. **Emma M. Krop:** Data curation, Visualization, Supervision, Writing - review & editing. **Ben Kew:** Formal analysis, Investigation. **Anwesha Sarkar:** Conceptualization, Methodology, Writing - review & editing, Visualization, Supervision, Funding acquisition.

## Declaration of competing interest

None.
