# Supplementary material for: Surface adsorption and lubrication properties of plant and dairy proteins: A comparative study
Source: Food Hydrocoll. 2021 Feb;111:106364. doi: 10.1016/j.foodhyd.2020.106364 (PMC7607376; doi:10.1016/j.foodhyd.2020.106364)
Supplement: Multimedia component 1 [file mmc1.docx]

**Supporting Information**

**Surface adsorption and lubrication properties of plant and dairy proteins: A comparative study**

Morfo Zembyla^1^, Evangelos Liamas^1^, Efren Andablo-Reyes^1^, Kewei Gu^1^, Emma M. Krop^1^, Ben Kew^1^, Anwesha Sarkar^1^*

^1^Food Colloids and Bioprocessing Group, School of Food Science and Nutrition, University of Leeds, Leeds, LS2 9JT, UK

*Corresponding author:

Dr. Anwesha Sarkar

Food Colloids and Bioprocessing Group,

School of Food Science and Nutrition,

University of Leeds, Leeds LS2 9JT, UK.

E-mail address: [A.Sarkar@leeds.ac.uk](mailto:A.Sarkar@leeds.ac.uk) (A. Sarkar).

**Table S1.** Final concentration, protein soluble fraction, size, PDI and ζ-potential of WPI and PPI dispersions at pH 6.8 under different treatments (without centrifugation, with centrifugation and/or after heat treatment (HT)). Samples with the same superscript letter do not differ significantly (*p > 0.05*) according to Tukey’s test.

| Samples | Final concentration (mg/mL) | Protein soluble fraction/ % | *D*_H_ (nm) | PDI | ζ-potential (mV) |
| --- | --- | --- | --- | --- | --- |
| 0.1 mg/mL un-centrifuged WPI | 0.1 ± 0.01 ^a^ | 95 ^c^ | 372.8 ± 92.8 | 0.54 ± 0.13 ^g^ | -20.7 ± 1.0 ^i^ |
| 0.1 mg/mL WPI centrifuged | 0.1 ± 0.02 ^a^ | 95 ^c^ | 217.8 ± 27.3 ^e^ | 0.42 ± 0.08 ^g^ | -20.2 ± 0.7 ^i^ |
| 0.1 mg/mL WPI centrifuged and HT | 0.09 ± 0.020 ^a^ | 94 ^c^ | 174.7 ± 34.9 ^e^ | 0.34 ± 0.02 ^g^ | -18.4 ± 1.0 ^i^ |
| 0.1 mg/mL un-centrifuged PPI | 0.09 ± 0.01 ^a^ | 87 | 3836.7 ± 213.9 | 1.64 ± 0.08 ^h^ | -19.6 ± 1.9 ^j^ |
| 0.5 mg/mL PPI centrifuged | 0.07 ± 0.01 ^b^ | 14.8 ^d^ | 259.0 ± 52.7 ^e^ | 0.55 ± 0.01 ^g^ | -21.0 ± 1.1 ^j^ |
| 0.5 mg/mL PPI centrifuged and HT | 0.07 ± 0.01 ^b^ | 13.4 ^d^ | 132.0 ± 4.0 ^f^ | 0.29 ± 0.01 ^g^ | -21.4 ± 3.1 ^j^ |

**Table S2.** Rate of adsorption of WPI and PPI (heated or not) on gold or PDMS surface.

| **Gold** | **Rate of adsorption (mg/m^2^/min)** |
| --- | --- |
| **Non-heat-treated WPI** | 4.3 |
| **Non-heat-treated PPI** | 2.1 |
| **WPI HT** | 4.9 |
| **PPI HT** | 2.6 |
| **PDMS** | **Rate of adsorption (mg/m^2^/min)** |
| **Non-heat-treated WPI** | 0.7 |
| **Non-heat-treated PPI** | 3.0 |
| **WPI HT** | 2.4 |
| **PPI HT** | 3.3 |


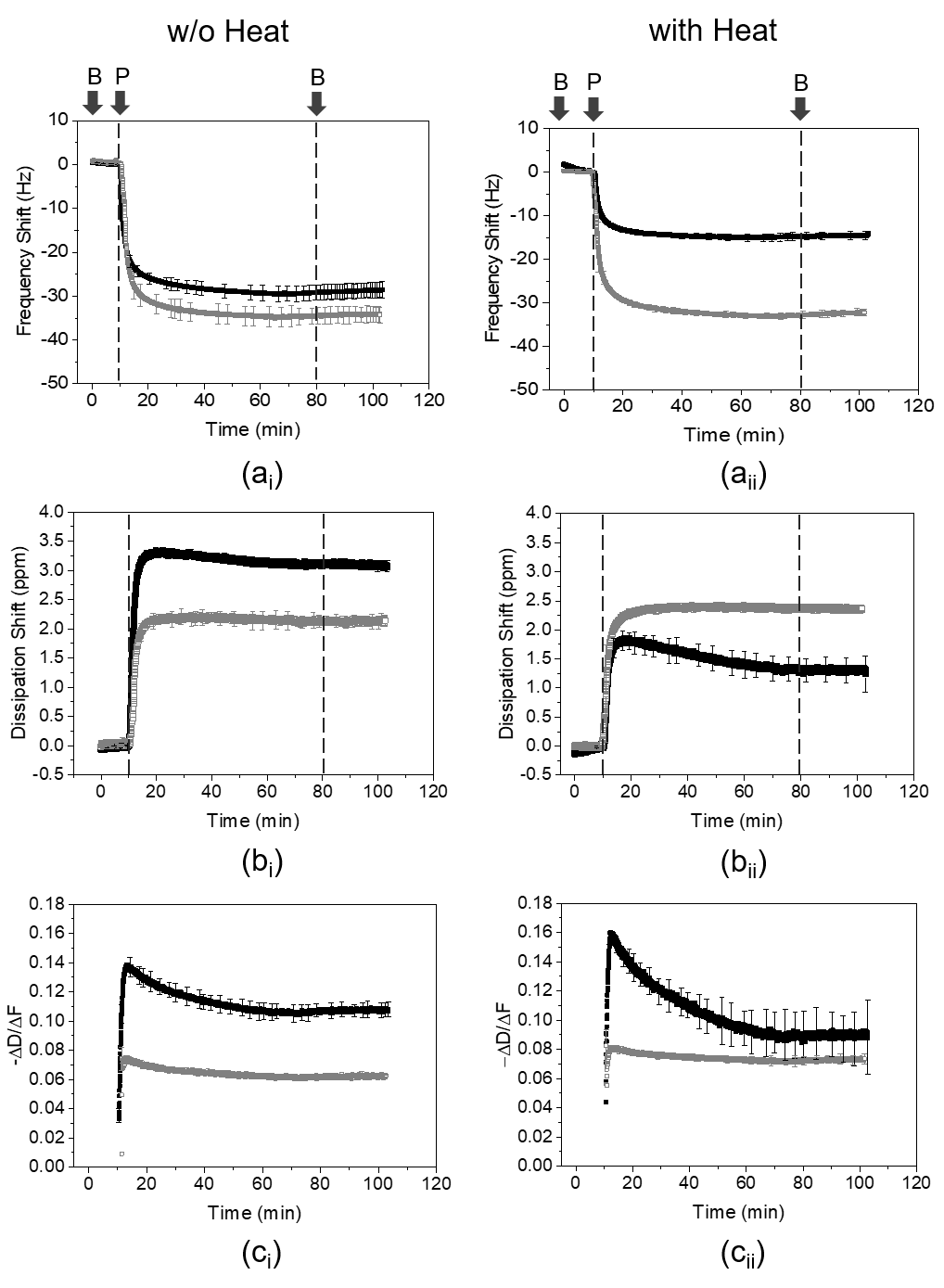


**Figure S1.** Frequency shift (a), dissipation shift (b) and *–ΔD/Δf* (c) obtained (5^th^ overtone shown), as a function of time, of 0.1 mg/mL WPI (■) and PPI (□) on gold surface without (i) or with (ii) HT at 90 ℃. B and P indicate addition of buffer and protein, respectively. Error bars represent standard deviations.


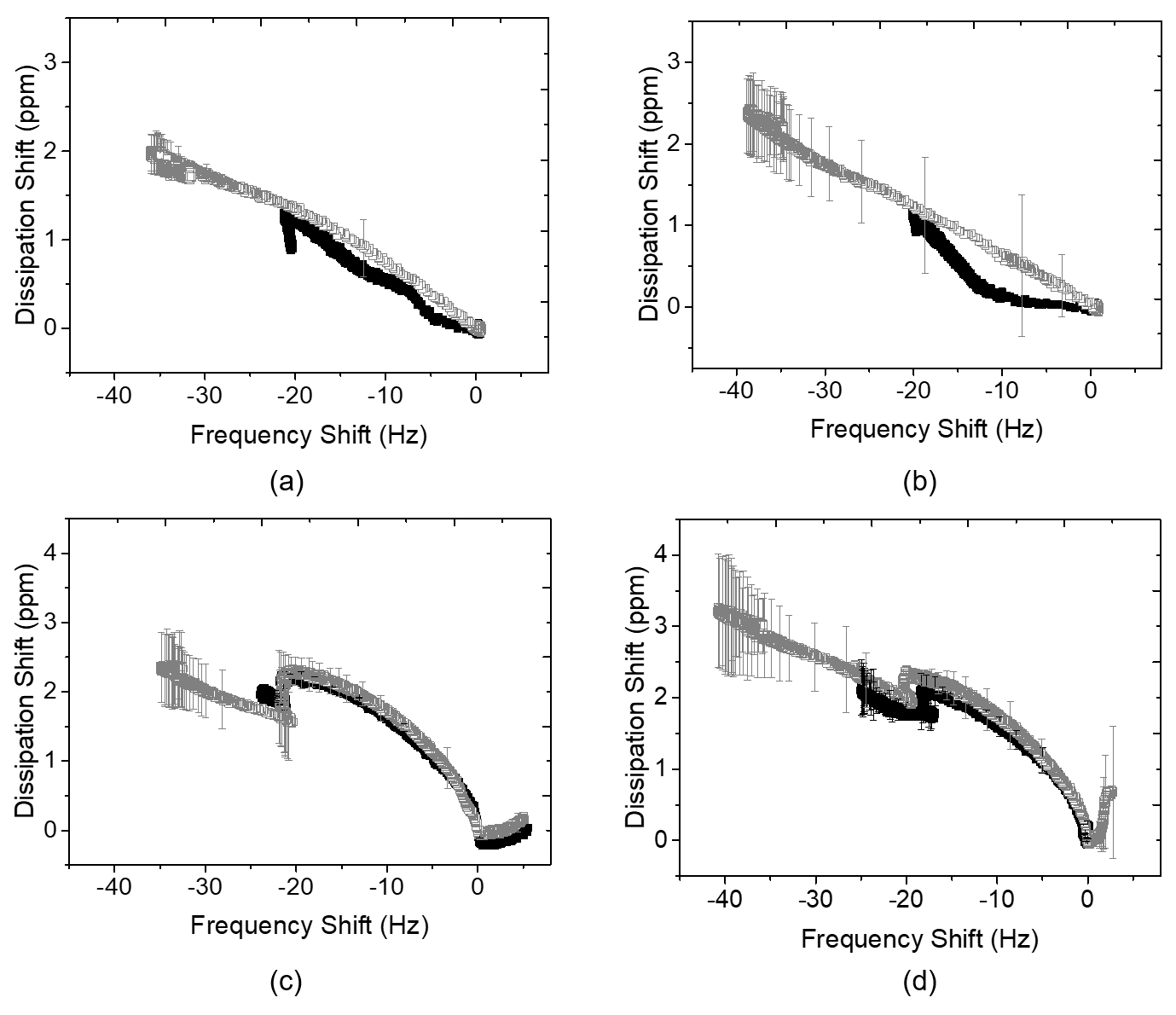


**Figure S2.** Dissipation shift against frequency shift for 0.1 mg/mL WPI against PPI (■, □ for WPI and PPI, respectively); (a) without HT, (b) with HT, (c) 1.0 mg/mL BSM adsorption prior to the protein adsorption without HT and (d) 1.0 mg/mL BSM adsorption prior to protein adsorption with HT on PDMS-coated surface. Error bars represent standard deviations.


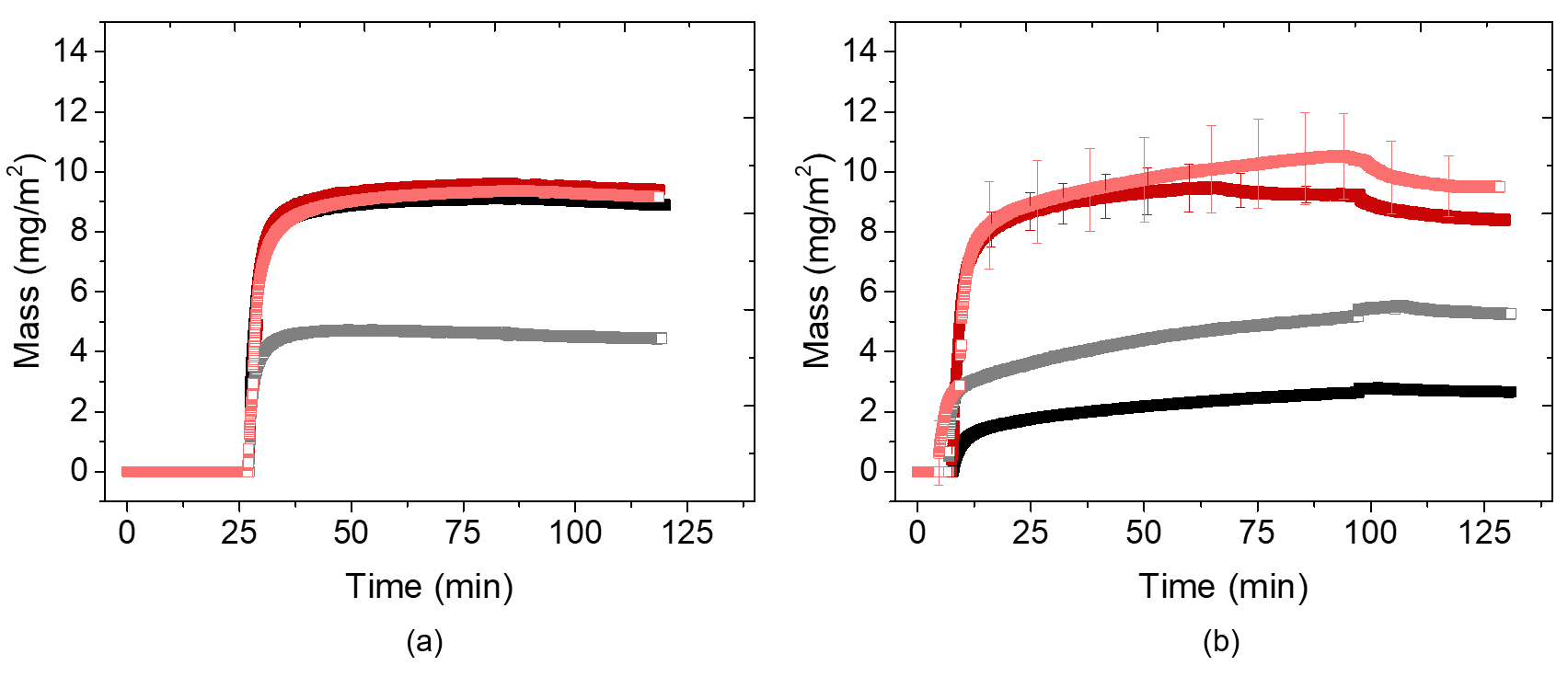


**Figure S3.** Adsorbed mass against time for 0.1 mg/mL WPI and PPI without HT (■, ■ for WPI and PPI, respectively) or with HT (□, □ for WPI and PPI, respectively) on (a) gold surface and (b) PDMS-coated surface. Error bars represent standard deviations.
